# Delayed school progression and mental health problems in adolescence: a population-based study in 10,803 adolescents

**DOI:** 10.1186/s12888-014-0244-5

**Published:** 2014-09-14

**Authors:** Wanda M Tempelaar, Christiaan P Otjes, Clothilde J Bun, Carolien M Plevier, Willemijn A van Gastel, James H MacCabe, René S Kahn, Marco PM Boks

**Affiliations:** Rudolf Magnus Institute of Neuroscience, Department of Psychiatry, University Medical Center Utrecht, Utrecht, The Netherlands; Community Health Service Midden-Nederland, Zeist, The Netherlands; Institute of Psychiatry, Department of Psychosis Studies, King’s College London, London, The United Kingdom

**Keywords:** Adolescence, Confounding, Delayed school progression, General mental health and SDQ

## Abstract

**Background:**

Accumulating evidence suggests that several adult mental disorders, particularly psychoses, are preceded by impairments in cognitive function, reflected in scholastic underachievement. This study investigates the association between scholastic underachievement and general mental health problems in adolescence, using delay in school progression as a marker of poor scholastic performance.

**Method:**

Cross-sectional secondary school survey comprising 10,803 adolescents. Participants completed the Strengths and Difficulties Questionnaire (SDQ) to assess mental health problems. The association of delayed school progression with the SDQ was investigated using logistic regression with SDQ as outcome and delayed school progression as primary exposure of interest while adjusting for socio-demographic characteristics, adverse life events, school-related factors, risk taking behaviour, healthy lifestyle and physical health.

**Results:**

Unadjusted analysis showed an association between delayed school progression and total mental health problems (OR 1.83, 95% CI 1.27 – 2.63) in adolescents. After adjusting for other risk factors (socio-demographic factors and life events) in a logistic regression model the association between delayed school progression en mental health problems was attenuated (OR 1.33, 95% CI 0.86 – 2.05).

**Conclusion:**

Delayed school progression is associated with general mental health problems in adolescence, but this relationship is heavily confounded by other factors. A causal relationship between impaired cognitive function such as poor scholastic performance and general mental health at adolescence is less likely and delayed school progression may merely be considered an indicator of risk for mental health problems.

**Electronic supplementary material:**

The online version of this article (doi:10.1186/s12888-014-0244-5) contains supplementary material, which is available to authorized users.

## Background

Mental health difficulties are both common and debilitating in childhood. About one-third of all children experience mental health problems that affect everyday functioning [1,2]. Without adequate treatment these symptoms often persist into adulthood [3]. Since approximately fifty percent of all lifetime mental disorders arise in the mid-teens [4], recent attention has focused on early detection and intervention during adolescence.

Several studies show that poor cognitive function or intellectual achievement is present before the onset of several mental disorders. Low intelligence coefficient (IQ) has predominantly been associated with later development of schizophrenia (reviewed in [5]). Similar associations have been found for other psychiatric illnesses such as severe depression [6] and anxiety disorders [7]. Indeed, some studies suggest that pre-morbid low intellectual functioning is related to overall risk of psychiatric disorders in adulthood [8,9]. Various mechanisms have been suggested to underlie this relationship, including the hypothesis that impairments in IQ or other cognitive functioning reflect neurodevelopmental changes which may predispose to psychiatric disorders [10,11].

Several studies have demonstrated associations between poor scholastic achievement and mental disorders during adolescence or adulthood [12-15]. Repeating a grade is previously used as an indicator of scholastic underachievement and is for instance related to the risk of schizophrenia and other psychoses [14]. However, few studies investigated poor school achievement as an independent risk factor for the development of mental health problems during adolescence [13]. We therefore studied the association between a delay in school progression and mental health problems in adolescents, and adjusted for potential confounders.

## Methods

We conducted a cross-sectional population-based study of adolescents attending secondary schools in The Netherlands. The study population was an unselected sample of 10,803 adolescents at secondary school (age 12–18) who were routinely surveyed by the Community Health Service Central Netherlands & Eemland (GGD).

Dutch secondary school has four main levels of education that prepare for further education. For the purpose of this study we divided the group in those who were preparing for polytechnic or academic training and those that were focussing on vocational training. The survey took place during autumn 2007 and was conducted alongside prevention programs in all secondary schools in the province of Utrecht, excluding the City of Utrecht. The region includes both urban and rural areas; about 34% of the study population lives in an urban area. Participation was determined at school level and 45 schools (71% of the schools invited) participated. The most common reasons for schools not to participate were lack of time, change in management, or participation in other research projects. 84% of all students in the participating schools completed the questionnaire, non-response was mostly due to absence because of illness or truancy.

Institutional Review Board approval was not required for this study since it was an anonymous questionnaire obtained for public health purposes.

### Measurements

All students were asked to complete an anonymous digital questionnaire in the classroom.

#### Mental health problems

Mental health problems were defined as a score in the clinical range of the Dutch translation of the self-reported Strengths and Difficulties Questionnaire (SDQ) [16]. The Dutch translation of the self-reported SDQ is validated [17]. The SDQ has a specificity of 94.6% (95% CI 94.1-95.1) and a sensitivity of 63.3% (95% CI 59.7-66.9) to identify adolescents with a psychiatric disorder [18]. The SDQ consists of 25 items on psychosocial attributes. Each item is scored on a 3-point scale with ‘not true’ , ‘somewhat true’ , and ‘certainly true’. These 25 items are divided into 5 subscales, each with 5 items: emotional symptoms, conduct problems, hyperactivity/ inattention, peer relationship problems, and pro-social behaviour. The summed score, excluding the items about pro-social behaviour, generates a total difficulties score, with a range of 0 to 40. The SDQ total difficulties score can be divided into normal (0–15), borderline (16–19) and clinical scores (20–40) [16]. To identify those with clinically relevant symptoms, the outcome was dichotomized into normal to borderline and clinical.

#### Poor school achievement

We used delayed school progression as marker of previous poor school achievement. A student was considered a poor school achiever when his age was at least 1.5 years above the mean age for his grade. This stringent cut-off allowed us to identify adolescents who had failed to progress as expected to the next grade on at least one occasion at any stage of primary or secondary school [19].

### Potential confounders

Potential confounders were determined based on the literature and availability in the school survey which assessed poor school achievement and mental health problems. Potential confounding factors were categorized into six different subgroups: socio-demographic factors, adverse life events, school-related factors, risk taking behaviour, health & lifestyle and miscellaneous factors. The content of the questionnaires can be found in the Additional file 1.

#### Socio-demographic factors

Several socio-demographic factors were measured: gender, age (defined as low: under 14 years old), immigrant status, marital status of parents and socioeconomic position [20] and urbanization [21].

#### Adverse life events

The following adverse life events were assessed using adolescents’ self-report: death of a first degree relative or another loved person, chronic disease or long lasting hospitalization of the adolescent himself or a first degree relative, parental alcohol problem or addiction, psychiatric disease in a first degree relative, parental divorce, domestic violence (between parents or victimizing the adolescent), molestation by someone other than the parent and sexual abuse [20].

#### School-related variables

The following school-related variables were measured: educational level at secondary school [13], bullying, victimization [22], school perception [23], truancy [24], perceived school safety [25], getting along with classmates and having close friends in school.

#### Risk taking behaviour

Substance use was assessed by questions about frequency of consumption of alcohol, tobacco, marijuana, other illicit drugs and addiction [26,27]. Sexual risk taking behaviour was measured by condom use, lifetime multiple sexual partners and a history of sexually transmitted diseases (STDs).

#### Health and lifestyle

Healthy lifestyle was measured by seven variables: physical exercise, regular breakfast [28], fruit and vegetable consumption, overweight (BMI according to age), weight perception, excessive television/computer usage, absence at school due to illness and use of painkillers.

#### Miscellaneous factors

Family care, future perspective on life and parental involvement.

### Data analyses

#### Missing data

The SDQ was complete for 11,291 adolescents (247 incomplete cases (2.1%) were excluded). Also, data were missing for multiple sexual partners (477 missing cases (4.2%). Because of the relatively small amount of missing data casewise exclusion of missing cases was performed. Furthermore, outliers with age older than 20 were excluded (N = 10) as was one outlier with total SDQ score above 36. In total, data from 10,803 cases were analyzed.

#### Statistical analysis

All data were analysed using SPSS 19.0 for Windows. Bivariate non-parametric correlations were performed to identify multicollinearity between different variables (Kendall’s τ > 0.8) [29]. As described above, potential confounders were selected based on literature. We used the large set of potential risk factors to perform a data-driven approach. Therefore, we divided the analysis in three different phases. First, we analyzed the crude unadjusted association between delayed school progression and current mental health problems. The crude odds ratio (OR) with 95% confidence interval (CI) was determined using logistic regression analysis. Secondly, covariates were tested as to whether they (1) were significantly correlated with both poor school achievement and mental health problems, and (2) altered the association between delayed school progression and mental health problems by at least ten percent [30]. The final step in the analysis was the composition of a predictive model using multivariate logistic regression adjusting for only the confounding factors that were obtained in step two of the analysis. Significance level was set as p = 0.05.

## Results

The sample consisted of 10,803 participants, 99% of the participants were between 12 and 18 years old (range: 10–19 years), with a mean age of 14.2 (SD = 1.6) and 49.8% male. Gender was equally distributed. Most participants were Dutch and living with both parents. 35.4 % of the pupils were identified as delayed in their school progression. Male gender (OR 1.40, 95% CI 1.15 – 1.69) was associated with delayed school progression. Adolescents with a non-Dutch ethnicity had higher odds for a delay in school progression (OR 2.77, OR 95% CI 2.27 – 3.73). Mental health problems, however, were equally distributed by age, gender and ethnicity. Bivariate analysis did not show multicollinearity between different variables. Table 1 shows the demographic characteristics of this population; information about other variables is added to the Additional file 1: Table S1. Both of these tables show the distribution of variables analysed in the whole sample and clinical SDQ group, plus their relation with mental health problems.

**Table 1:** **The distribution of different demographic variables in the full sample, the clinical SDQ group and their association with clinical SDQ**

	**Full sample**	**Clinical SDQ**	**Association SDQ**
Variables	N (%)	N (%)	OR (95% CI)
Male gender	5378 (49.8)	260 (53.3)	1.16 (0.97-1.39)
Age (mean, range)	14.17 (10–19)	14.19 (10–18)	1.01 (0.95-1.07)
Non-Dutch ethnicity	2091 (19.4)	93(19.1)	0.98 (0.78-1.24)
Not living with both parents	2390 (22.1)	150 (30.7)	1.60 (1.31-1.95)*
Low SES^	929 (8.6)	95 (19.5)	2.75 (2.17-3.48)*
Vocational level of education (missing 148)	5538 (52.0)	327 (68.1)	2.04 (1.67-2.48)*
Urbanization (missing 1491)			
● Less than 1000 (ref.)	3314 (35.6)	146 (36.9)	…
● 1000 - 1499	4054 (43.5)	166 (41.9)	0.93 (0.74-1.16)
● 1500 - 2499	1859 (20.0)	81 (20.5)	0.99 (0.75-1.31)
● 2500 or more	85 (.9)	3 (.8)	0.79 (0.25-2.54)

*significant associations at p < 0.05.

°SDQ: Strengths and Difficulties Questionnaire.

^SES: SocioEconomic Status.

Analysis of potential confounders yielded a subgroup of variables which were added to the logistic models. (We selected only those confounders that were correlated with delayed school progression and SDQ and altered the association between delayed school progression and SDQ). These covariates were mainly adverse life events: sexual abuse, domestic violence and parental problems with alcohol or addiction. Other confounders were perceived problems with money or income and irregular breakfast. Since many control variables were related to age, gender and ethnicity, these factors were also included in the prediction model.

Table 2 shows both the unadjusted and the adjusted prediction model. The unadjusted model consists of the crude association between delayed school progression and mental health problems (OR 1.83, 95% CI 1.27-2.62). In the adjusted model all the potential confounding factors were entered together. In the adjusted logistic regression model the association between delayed school progression and clinical SDQ was not statistically significant (OR 1.38, 95% CI 0.91-2.10). Since the association was not longer significant, analysis of factors that might also act as mediating factors was redundant.

**Table 2:** **Prediction models showing delayed school progression as a predictor for mental health problems**

	**Unadjusted model**	**Adjusted model confounders**
Predictor	Odds ratio (95% CI)	Odds ratio (95% CI)
Delayed school progression	1.83 (1.27 - 2.63)*	1.38 (0.91-2.10)
Socio-demographic factors:		
Low age		0.91 (0.85-0.97)*
Male gender		1.34 (1.11-1.63)*
Dutch ethnicity		0.69 (0.54-0.89)*
Economic problems		3.58 (2.85-4.51)*
Irregular breakfast		2.09 (1.68-2.61)*
Adverse life events:		
Sexual abuse		1.92 (1.32-2.80)*
Molestation by parents		2.10 (1.43-3.08)*
Violence between parents		1.55 (1.06-2.27)*
Parental problems with alcohol/addiction		1.90 (1.37-2.64)*

*significant associations at p < 0.05.

## Discussion

In this cross-sectional study we investigated the association between scholastic achievement and current mental health problems in adolescents. We demonstrated that delayed school progression, used as a proxy for poor school achievement, was associated with adolescent mental health problems. After adjusting for potential confounding factors the association was no longer significant. These findings indicate a possible role for delayed school progression as a risk marker for mental health problems at adolescence, but did not provide evidence that delayed school progression is a causal risk factor for mental health problems. However this does not rule out the possibility that school performance may be associated with specific psychiatric outcomes such as schizophrenia.

The factors that we identified as confounders are important to consider in further studies.

### Strengths and limitations

The main strengths of this study were the large population-based sample with adolescents of both genders and the richness of the data, particularly on potential confounders. Furthermore, a delay in school progression is an objective measure and not subject to recall bias. The response rate was generally good. Moreover, the association between delayed school progression and mental health problems might have been stronger when non-responders would have joined the study since truants or frequently ill students are probably more likely to have high SDQ scores and problems with scholastic achievement.

This study also has limitations. Delay in school progression was a rather crude proxy for poor school performance and children with delayed school progression probably constituted a heterogeneous group, since the delay could have been the result of enrolling in primary school at a relatively old age or language problems. We reduced this problem considerably by adjusting for covariates, such as ethnicity, and by picking a stringent cut-off point for delayed school progression to include only children with a definite history of scholastic underperformance. Another limitation was the self-reported SDQ as an outcome measure. Although validated and used widely as a measure for mental health problems, complementary information from teachers and parents would improve the sensitivity and specificity. Furthermore, the prevalence of mental health problems in our study population was low compared with international studies using the SDQ [18]. Dutch children have a tendency to score lower on the SDQ than UK children; about 6% of all Dutch children score above the UK cut-off point [2] and in our sample 4.5% of the participants scored in the clinical range. Additionally, the included children were aged between 10 and 19 years old (99% between 12 and 18 years) and mental health problems may differ between the younger and older participants. This study was also limited by its cross-sectional design. Delay in school progression and mental health problems were measured at the same moment, thus we cannot rule out that mental health problems could have preceded the delay in school progression earlier in youth. However, previous prospective studies on educational performance showed that poor educational attainment often predicts development of psychiatric disorders [12-15].

### Interpretation of findings

Contrary to most previous studies addressing poor educational achievement and psychiatric problems, we found no association after adjusting for confounders. This finding is in contrast to previous studies which have shown associations between poor school performance and later psychosis. It is possible that the adolescent age is too early to detect the effects of neurobiological changes on mental health. Furthermore, we focused on general mental health problems, instead of the development of specific (mainly psychotic) disorders in the majority of previous studies.

In addition, poor school achievement can be seen as an event that might mediate the impact of other risk factors for mental health problems. Thus, poor school achievement might function as a trigger for alterations in the causal pathway of genetic and environmental factors underlying neurobiological changes leading to mental disorders.

Lastly, most studies used other measures of poor school performance, like school level [13], literacy skills [12] and school grades [14,15]. These measures may be more related to intellectual functioning than a delay in school progression because the latter is more affected by other aspects such as social and emotional development during school-age. Thus, further research is needed to examine the potential role of school performance as an intermediate factor for different risk factors of mental health problems and longitudinal effects of poor school performance.

## Conclusion

Overall our results showed that delayed school progression is a risk marker of mental health problems in school aged children, but that this association is largely explained by confounding. This is consistent with the view that poor school performance is a risk indicator for particular psychiatric disorders but not for general mental health problems in adolescents.

## Additional file

Additional file 1:
**Detailed content of the questionnaire and Supplementary table: distribution of all variables in the sample, clinical SDQ group and their association with SDQ.**
