# Supplementary material for: Delayed school progression and mental health problems in adolescence: a population-based study in 10,803 adolescents
Source: BMC Psychiatry. 2014 Sep 14;14:244. doi: 10.1186/s12888-014-0244-5 (PMC4177435; doi:10.1186/s12888-014-0244-5)
Supplement: Additional file 1: — Detailed content of the questionnaire and Supplementary table: distribution of all variables in the sample, clinical SDQ group and their association with SDQ. [file 12888_2014_244_MOESM1_ESM.pdf]

Appendix to "Delayed school progression and mental health problems in adolescence: a population-based study in 10,803 adolescents."

Wanda Tempelaar [1], Christiaan Otjes[1], Clothilde Bun[2], Carolien Plevier[2], Willemijn van Gastel[1], James MacCabe[3], René Kahn[1], Marco Boks[1]

Details of the full assessment by domain:

Socio-demographic factors

Immigrant status: 'both parents Dutch' or 'one or both parents born abroad'.

Marital status parents: 'living with both parents' or 'other'.

Socioeconomic position: 'no, parents never experienced problems with money and/or income' or 'yes, experienced or experiencing right now'.

Urbanization: measured in four levels of urbanization according to the Dutch Central Bureau of Statistics [31].

Adverse life events

All adverse life event factors were answered on a 2-point scale: 'Yes experienced or experiencing now' or 'no, never experienced'.

School-related factors

Educational level at secondary school: ‘vocational training’ or ‘polytechnic or academic training’.

Bullying: participated in bullying of other student in the last three months: ‘yes’ or ‘no’

Victimized: was victim of bullying during in the last three months: ‘yes’ or ‘no’

School perception, dichotomized in: ‘(very) nice’ or ‘okay, not nice and terrible’

Truancy: more than 3 hours last 4 weeks: ‘yes’ or ‘no’.

Getting along with classmates, dichotomized in: ‘almost none’ or ‘almost all, more than half and some’.

Good friends in school, dichotomized in: ‘no good friends’ or ‘one, some and many good friend’

Feeling unsafe at school, dichotomized in: ’never or sometimes’ or ‘(very) often’

Risk taking behaviour

Alcohol: weekly alcohol usage: ‘yes’ or ‘no’

Tobacco: daily smoking: ‘yes’ or ‘no’

Marijuana: marijuana consumption last month: ‘yes’ or ‘no’

Hard drugs: hard drugs consumption last month: ‘yes’ or ‘no’

Perceived difficulties with alcohol or addiction ever or perceives difficulties right now: ‘yes’ or ‘no’

Condom usage: used a condom during last sexual intercourse: ‘yes’ or ‘no’

Multiple sexual partners: four or more different sexual partners lifetime: ‘yes’ or ‘no’

Having a history of sexually transmitted disease (STD): ‘yes’ or ‘no’

Health and lifestyle factors

Physical exercise according to movement standard as defined by Dutch guideline healthy movement [32]: ‘yes’ or ‘no’.

Regular breakfast (at least six days a week): ‘yes’ or ‘no’.

Fruit consumption as defined by Dutch health standard [33]: ‘yes’ or ‘no’.

Daily vegetables consumption: ‘yes’ or ‘no’.

Weight perception: ‘way too thin’, ‘bit thin’, ‘normal’, ‘bit thick’, ‘way too thick’.

Excessive television usage: more than two hours a day or more: ‘yes’ or ‘no’.

Excessive pc usage: more than two hours a day or more: ‘yes’ or ‘no’.

Absence at school due to illness: at least one day or more last four weeks: ‘yes’ or ‘no’.

Frequent pain killer use: more than one every week in the past month: ‘yes’ or ‘no’.

Miscellaneous factors

Future perspective: the upcoming five year will be: ‘(very) good - not good, not bad’ or ‘ (very) bad’.

Family care: one or more of the following: ‘care for brothers and sisters; like dressing, feeding, bringing to school and babysitting’ or ‘taking care of things; like going to pharmacy,

coming to doctor/hospital or translating' or 'emotional support giving to sick parent or family member; like talking, listening and providing comfort'.

Parental involvement is measured in two ways: (1) Often joining parent teachers meetings (PTM), dichotomized in: '(almost) always' or 'sometimes and (almost) never) and (2) parental help with homework both dichotomized in: 'yes' or 'no'.

Supplementary table: Distribution of all variables in the sample, clinical SDQ group and their association with SDQ.

Variables	Full Sample<br>N= 10,803<br>N (%)	Clinical SDQ<br>N= 488<br>N (%)	Association SDQ<br>OR (95% CI)
Family care task	748 (6.9)	80 (16.4)	2.83 (2.20-3.64)*
Poor future perspective<br>(N = 10779; 486)	191 (1.8)	83 (17.1)	19.42 (14.35-26.30)*
Poor parent involvement			
• Homework (N = 10802; 0)	632 (5.9)	83 (17.0)	3.65 (2.84-4.69)*
• PTM (N = 10802; 0)	3030 (28.1)	192 (39.3)	1.71 (1.42-2.06)*
Loss of a relative	7389 (68.4)	352 (72.1)	1.21 (0.99-1.48)
Chronic disease / long hospitalization self	1893 (17.5)	130 (26.6)	1.76 (1.43-2.17)*
Chronic disease / long hospitalization relative or loved person	4303 (39.8)	245 (50.2)	1.56 (1.30-1.87)*
Parental addiction	467 (4.3)	68 (13.9)	4.02 (3.06-5.30)*
Psychiatric disease in one or both parents	636 (5.9)	77 (15.8)	3.27 (2.53-4.23)*
Psychiatric disease in brother or sister	550 (5.1)	56 (11.5)	2.58 (1.92-3.45)*
Parental divorce	2038 (18.9)	134 (27.5)	1.67 (1.36-2.05)*
Domestic violence between parents	380 (3.5)	57 (11.7)	4.09 (3.04-5.51)*
Molestation by parent(s)	331 (3.1)	58 (11.9)	4.96 (3.68-6.69)*
Molestation by other adult	244 (2.3)	44 (9.0)	5.01 (3.57-7.04)*
Sexual abuse	356 (3.3)	53 (10.9)	4.03 (2.96-5.47)*
Bullying (N = 10802; 0)	2987 (27.7)	240 (49.2)	2.67 (2.22-3.20)*
Victimized (N = 10802; 0)	1961 (18.2)	222 (45.5)	4.12 (3.42-4.95)*
Poor school perception	4566 (42.3)	345 (71.1)	3.56 (2.91-4.34)*

Frequent truancy	753 (7.0)	108 (22.1)	4.26 (3.39-5.35)*
Feeling unsafe at school (N = 10802; 0)	246 (2.3)	74 (15.2)	10.54 (7.89-14.08)*
Not getting along with classmates (N = 10802)	158 (1.5)	48 (9.8)	10.12 (7.12-14.39)*
No good friends in school (N =10802; 0)	130 (1.2)	29 (5.9)	6.39 (4.18-9.76)*
Weekly alcohol use	1933 (17.9)	168 (34.4)	2.54 (2.10-3.09)*
Marijuana use last month	676 (6.3)	96 (19.7)	4.11 (3.24-5.22)*
Daily smoking	786 (7.3)	701 (6.8)	2.89 (2.26-3.70)*
Drinking problem/addiction	338 (3.1)	268 (2.6)	6.28 (4.74-8.31)*
Hard drug use last month	129 (1.2)	88 (.9)	10.66 (7.27-15.63)*
Condom used last sexual intercourse (N=1743; 173)	566 (32.5)	484 (30.8)	2.02 (1.47-2.78)*
Multiple sexual partners lifetime	393 (3.6)	329 (3.2)	4.52 (3.45-6.09)*
History of STD	77 (.7)	54 (.5)	9.40 (5.72-15.45)*
Insufficient physical exercise	1529 (14.2)	99 (20.3)	1.58 (1.26-1.99)*
Insufficient fruit consumption	2235 (20.7)	102 (20.9)	1.01 (0.81-1.27)
Insufficient vegetables consumption	6932 (64.2)	322 (66.0)	1.09 (0.90-1.32)
Non-regular breakfast	1626 (15.1)	145 (29.7)	2.52 (2.06-3.09)*
Overweight (N = 10605; 479)	839 (7.9)	55 (11.5)	1.55 (1.16-2.07)*
Perception of overweight (N = 10802; 0)	3133 (29.0)	219 (44.9)	2.07 (1.72-2.48)*
Excessive computer usage	987 (9.1)	166 (34.0)	2.39 (1.99-2.87)*
Excessive television usage	3082 (28.5)	217 (44.5)	2.08 (1.73-2.50)*
Frequent painkiller usage (N = 10802; 0)	887 (8.2)	109 (22.3)	3.53 (2.81-4.42)*
School absence due to illness	3802 (35.2)	3553 (34.4)	1.98 (1.65-2.38)*

* *significant associations at p<0.05*

All variables were analysed on full sample size unless otherwise denoted in the first column. In case of smaller sample size, the number of missing values in the clinical SDQ group can be found in the left column directly after the sample size.

References Appendix

31. van Os J, Hanssen M, Bijl RV, Vollebergh W: **Prevalence of psychotic disorder and community level of psychotic symptoms: an urban-rural comparison.** *Arch Gen Psychiatry* 2001, **58**:663-668.

32. Kemper HGC, Ooijendijk WTM, Stiggelbout M: **Consensus over Nederlandse Norm voor Gezond Bewegen**. *Tijdschrift voor Sociale Gezondheidszorg* 2000, **78**:180-183.

33. Hulshof KFAM, Ocke MC, van Rossum CTM, Buurma-Rethans EJM, Brants HAM, Drijvers JJMM, **Resultaten van de Voedselconsumptiepeiling** *2003. 2004;* RIVM-rapport nr. 350030002. Bilthoven: RIVM.
